# Test performance of a commercial cryptococcal antigen lateral flow assay: a retrospective and prospective study at five Canadian sites

**DOI:** 10.1128/jcm.01438-25

**Published:** 2026-04-22

**Authors:** Catherine A. Hogan, LingHui David Su, Jonathan Laley, Erin Carruthers, Adrienne Wong, Susan Roman, Kulvinder Mannan, Pamela Kibsey, Balbir Jaswal, Bing Wang, Krysta Grant, Caroline Sheitoyan-Pesant, Navdeep Chahil, Marthe K. Charles, Vincent Tang, Constance Byrne, David M. Goldfarb, Muhammad G. Morshed

**Affiliations:** 1 British Columbia Centre for Disease Control Public Health Laboratory, Vancouver, British Columbia, Canada; 2 Department of Pathology and Laboratory Medicine, University of British Columbia 8166, Vancouver, British Columbia, Canada; 3 Fraser Health 27355 , Surrey, British Columbia, Canada; 4 Division of Microbiology and Molecular Diagnostics, Royal Jubilee Hospital 60292, Victoria, British Columbia, Canada; 5 Division of Medical Microbiology, Kelowna General Hospital 59140, Kelowna, British Columbia, Canada; 6 Centre Hospitalier Universitaire Dr-Georges-L.-Dumont 10012, Moncton, New Brunswick, Canada; 7 Division of Medical Microbiology and Infection Control, Department of Pathology and Laboratory Medicine, Vancouver General Hospital, Vancouver Coastal Health 25469, Vancouver, British Columbia, Canada; 8 Provincial Health Services Authority 8145, Vancouver, British Columbia, Canada; 9 BC Children’s Hospital and Women’s Hospital and Health Centre 8163, Vancouver, British Columbia, Canada; University of Utah, Salt Lake City, Utah, USA

**Keywords:** *Cryptococcus*, lateral flow assay, diagnosis, fungal

## Abstract

Cryptococcal antigen (CrAg) lateral flow assay (LFA) enables the rapid and sensitive detection of cryptococcal infection. The FungiXpert CrAg LFA is a Health Canada-licensed product that currently lacks evaluation in the North American setting. This retrospective and prospective study across five Canadian laboratories aimed to evaluate the FungiXpert CrAg LFA compared to the IMMY CrAg LFA for serum and cerebrospinal fluid (CSF) samples. Positive percent agreement (PPA) and negative percent agreement (NPA), precision, and cross-reactivity were assessed. A retrospective data review was performed for demographics, clinical data, and additional diagnostic testing results. A total of 141 serum samples and 93 CSF samples from 192 individuals were included. In the overall retrospective cohort, the FungiXpert CrAg LFA demonstrated qualitative detection PPA of 90.2% (95% confidence interval [95% CI], 87.3%–93.1%), and NPA of 100% (95% CI, 97.7%–100%). By sample type, the CSF PPA was 93.9% (95% CI, 79.8%–99.3%), and the serum PPA was 90.7% (95% CI, 79.7%–96.9%). The prospective cohort consisted of 58 samples, including two serum samples that were positive; of these, one was a low titer (1:1) and missed by FungiXpert. Both assays showed cross-reactivity with *Trichosporon asahii*. Inter-assay precision evaluation highlighted substantial variability in the FungiXpert CrAg LFA serum testing. These findings suggest similar performance of the FungiXpert CrAg LFA on CSF samples, but lower sensitivity on serum samples and greater lot-to-lot variability compared to IMMY CrAg LFA that should be considered in clinical implementation decision-making. In this Canadian multicenter study, the FungiXpert CrAg LFA demonstrated similar performance on CSF samples but lower sensitivity on serum samples compared to the IMMY CrAg LFA.

**IMPORTANCE:**

This multicenter Canadian evaluation provides the first North American performance data for the Health Canada-licensed FungiXpert cryptococcal antigen lateral flow assay. While performance on cerebrospinal fluid was comparable to the reference IMMY assay, reduced sensitivity for low-titer serum samples and greater inter-assay variability highlight important considerations for clinical implementation and assay selection.

## INTRODUCTION

Cryptococcosis refers to clinical disease caused by any of the seven pathogenic *Cryptococcus* species that form part of the *Cryptococcus gattii* sensu lato (s.l.) and *Cryptococcus neoformans* s.l. species complexes (1). Meningitis, pulmonary, and cutaneous involvement represent the most common clinical presentations, with the poorest prognosis associated with central nervous system and disseminated disease (2). *Cryptococcus neoformans* s.l. accounts for over 99% of cases globally and disproportionately affects immunocompromised hosts, including persons living with HIV and solid organ transplant recipients (3, 4). *Cryptococcus deuterogattii* has emerged over the last two decades in the Pacific Northwest of Canada and the United States and has demonstrated a capacity to infect immunocompetent hosts or individuals presumed to be immunocompetent and later found to harbor specific immunologic disorder, such as antibodies against granulocyte-macrophage colony-stimulating factor (4–8). The mortality rate of cryptococcal meningitis reaches 20% (2), and timely and accurate diagnosis is crucial to enable early effective treatment and reduce mortality (9, 10).

The cryptococcal antigen (CrAg) lateral flow assay (LFA) is an immunochromatographic assay for the qualitative and semi-quantitative detection of cryptococcal capsular polysaccharide antigen directly from serum and cerebrospinal fluid (CSF) samples. Based on data generated using several commercially available CrAg assays, this testing approach is rapid and highly accurate in HIV-infected (11, 12) and uninfected individuals (13, 14). The CrAg LFA (IMMY Diagnostics, Norman, Oklahoma) was approved by the U.S. Food and Drug Administration (FDA) in 2011 and is one of the main assays used globally; however, it is not currently licensed by Health Canada. Another assay, the FungiXpert cryptococcal capsular polysaccharide detection K-set (Genobio Pharmaceutical, Tianjin, China), entered the market more recently and received Health Canada licensure in March 2023; however, it is not currently licensed by the U.S. FDA. Limited studies have been performed to evaluate the performance of the FungiXpert LFA, and data generated in the North American context are lacking (15). Latex agglutination (LA) for CrAg detection is also performed in certain settings, particularly where the LFA has not been validated, and there are currently no published data on test performance compared to the FungiXpert CrAg LFA.

This study aimed to evaluate the test performance of the FungiXpert CrAg LFA strip format compared to the IMMY CrAg LFA for serum and CSF samples across five Canadian sites. In addition, the performance of the FungiXpert CrAg LFA was evaluated against LA for a single site, where it was performed as part of routine testing.

## MATERIALS AND METHODS

### Study design

Five healthcare facilities across two Canadian provinces participated in the study: four in British Columbia (BC) and one in New Brunswick (NB). The BC sites included the British Columbia Centre for Disease Control (BCCDC) Public Health Laboratory, which tests samples across the province; Surrey Memorial Hospital (SMH) from the Fraser Health Authority (FHA), Kelowna General Hospital (KGH) from Interior Health Authority (IHA), and the Royal Jubilee Hospital (RJH) located in Victoria, BC from Island Health (IH). These four facilities are collectively referred to as the BC cohort. The NB site included a single site: the Dr. Georges-L.-Dumont University Hospital Centre (GLD-UHC). This study evaluated the FungiXpert CrAg LFA against the IMMY CrAg LFA. These two CrAg LFA assays were selected for evaluation given that they are the most commonly used in the Canadian setting. Routine CrAg testing is performed with the IMMY CrAg LFA in the BC cohort, and with the cryptococcal LA for the GLD-UHC site. Given this, the BCCDC performed IMMY CrAg LFA and repeated FungiXpert CrAg LFA testing for the NB site. The IMMY CrAg LFA kits were secured through the Health Canada Special Access Program (16), as this assay is not currently licensed by Health Canada. Additionally, the comparison for the NB site incorporated LA in addition to the other two CrAg LFAs described above. Testing was performed per manufacturer recommendations for both CrAg LFA assays (17, 18). For semi-quantitative testing, samples were diluted across the range of manufacturer-recommended ratios as follows: 1:1 (undiluted), 1:5, 1:10, 1:20, 1:40, 1:80, 1:160, 1:320, 1:640, 1:1,280, and 1:2,560. CrAg test results were compared to identification by complementary standard-of-care diagnostic methods, including histopathology, fungal culture, matrix-assisted laser desorption ionization–time of flight mass spectrometry, and internal transcribed spacer (ITS) rRNA gene sequencing, when available. Furthermore, because molecular genotyping to species or lineage level was available for only a subset of *Cryptococcus* isolates, results in this study are reported as sensu lato*.*

#### Retrospective cohort

Frozen archived samples for the retrospective cohort were identified from all individuals who underwent clinical testing for CrAg using LFA or LA on serum or CSF at the five participating Canadian laboratories between July 2017 and June 2024, and selected to represent a comprehensive range of CrAg titers. All CrAg-positive samples from unique collection dates per individual with a residual volume of at least 400 μL were included in the study. In addition, a convenience set of negative samples for serum and CSF was included at each site based on sample availability. All retrospective samples were stored at −20°C until testing.

For the BC retrospective cohort, historical IMMY CrAg LFA result, titer and composite fungal testing results were compared with the new FungiXpert CrAg LFA result. In addition, for positive samples with sufficient residual sample volume, repeat testing by IMMY was performed. Two sites (FHA and IHA) performed qualitative CrAg LFA testing only; all other sites performed both qualitative and semi-quantitative testing. Testing was performed on site at each participating facility, except for NB, where only the FungiXpert CrAg LFA testing was conducted locally based on routine testing availability. Once the local testing was complete, the sample was then forwarded to the BCCDC for the IMMY CrAg LFA testing, where repeat FungiXpert CrAg LFA testing was performed. For all samples, if discrepancies were identified between the FungiXpert and IMMY results, repeat IMMY testing was performed if sufficient residual sample was available.

#### Prospective cohort

Prospective testing was performed at a single site, the BCCDC. For a 1-month period from April to May 2024, all serum and CSF samples requiring CrAg testing were tested in parallel using both the IMMY CrAg LFA assay and the FungiXpert CrAg LFA assay.

Further subgroup analyses were performed on discordant samples on qualitative interpretation, or with semi-quantitative results differing by two or greater doubling dilutions.

### Analytical performance evaluation

To assess for potential prozone phenomenon, a sample with a documented titer of ≥1:1,280 on IMMY CrAg LFA was re-tested in parallel with both the IMMY and FungiXpert CrAg LFA at the BCCDC. Cross-reactivity was assessed based on a distribution of samples positive for bacterial, fungal, and viral infections and from individuals with a confirmed alternative non-infectious diagnosis. Assessment of precision was performed at two sites, the BCCDC and FHA, based on sample availability. Intra-assay precision of the FungiXpert CrAg LFA was assessed by testing once daily over three consecutive days using reagents with the same lot number. Inter-assay precision of the FungiXpert CrAg LFA was assessed by testing in triplicate over 2 days using two different reagent lots. In addition, a limited inter-assay precision assessment of the IMMY CrAg LFA was performed by testing samples on a single day using two different reagent lots.

### Data analysis

Statistical analysis and data visualization were performed using Python 3.13 with the pandas 2.2.3, matplotlib 3.9.2, and SciPy 1.14.1 libraries. Qualitative data analysis was performed by calculating the overall percent agreement (OPA), positive percent agreement (PPA), negative percent agreement (NPA), and Cohen’s kappa between the assays, with 95% confidence intervals (CIs) determined by the Clopper–Pearson method (19). Semi-quantitative data analysis was performed by comparing the natural log-transformed inverse CrAg titers between the two CrAg LFAs. We performed a chart review for age, sex, immunocompromised host status, disease presentation, and results from orthogonal *Cryptococcus* testing, including CSF microscopy, fungal culture, broad-range fungal sequencing, and histopathology.

## RESULTS

**Fig 1:**
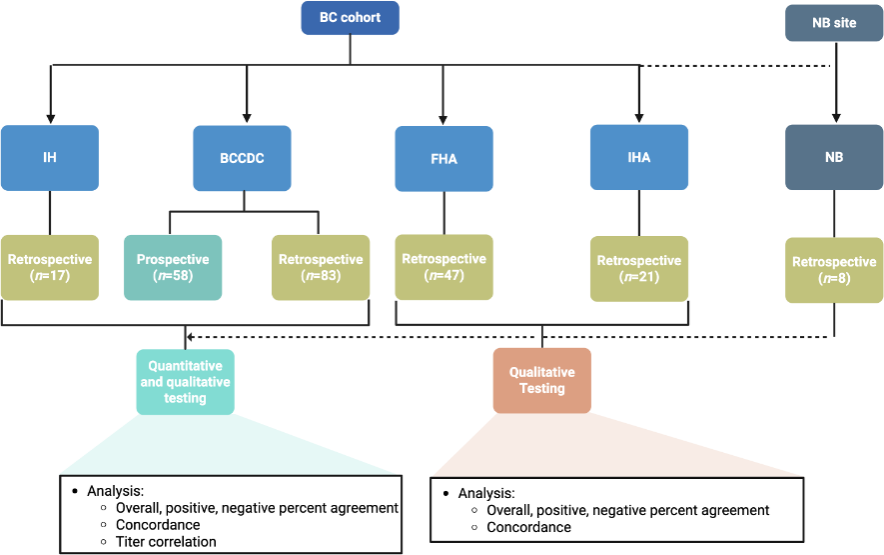
Overall study flowchart.

A total of 234 samples from 192 individuals were included in the study, including 176 samples in the retrospective cohort (88 positive, 88 negative) and 58 samples in the prospective cohort (2 positive, 56 negative) (Table 1; Fig. 1). Of the 192 individuals included, 28 contributed multiple samples (CSF and serum [*n* = 13], serum only [*n* = 8], and CSF only [*n* = 7]), and 164 contributed a single sample (serum only [*n* = 104], CSF only [*n* = 60]). Most samples were serum (59.0%), and the remainder were CSF (41.0%). A total of 90 individuals were female (46.9%), and the median age was 61 years (interquartile range [IQR], 45–72 years). Among the 59 individuals with cryptococcal infection, a total of 34 (57.6%) were immunocompromised, including 3 (5.1%) with HIV infection (Table 2). The most common site of infection was disseminated disease (as defined by two or more sites of infection) in 22 (37.3%), followed by isolated pulmonary in 17 (28.8%) and isolated CNS involvement in 14 (23.7%). Among individuals with culture-confirmed disease, 22 (61.1%) were *Cryptococcus neoformans* s.l. complex and 14 (38.9%) were *Cryptococcus gattii* s.l. complex.

**TABLE 1:** Distribution of serum and CSF samples included in the study for the five participating sites^*b*^

Site	Retrospective				Prospective				Total (%)
	Serum		CSF		Serum		CSF		
	Positive	Negative	Positive	Negative	Positive	Negative	Positive	Negative	
BC Centre for Disease Control (BCCDC)	24	20	18	21	2	48	0	8	141 (60.3)
Surrey Memorial Hospital (FHA)	20	3	7	17	0	0	0	0	47 (20.1)
Kelowna General Hospital (IHA)	0	9	0	12	0	0	0	0	21 (9.0)
Royal Jubilee Hospital (IH)	5	4	6	2	0	0	0	0	17 (7.3)
Dr. Georges-L.-Dumont University Hospital Centre^*a*^ (NB)	6	0	2	0	0	0	0	0	8 (3.4)
Total	55	36	33	52	2	48	0	8	234

^*a*^ All eight samples from this site were also included for CrAg latex agglutination testing.

^*b*^ BC: British Columbia; CSF: cerebrospinal fluid; FHA: Fraser Health Authority; LFA: lateral flow assay; NB: New Brunswick; IH: Island Health; IHA: Interior Health Authority.

**TABLE 2:** Basic demographic and clinical characteristics of the 59 individuals with cryptococcal infection included in the study^*a*^

		Retrospective^*d*^				Prospective^*e*^	Total (*n* = 59)
		BCCDC (*n* = 29)	FHA (*n* = 17)	IH (*n* = 8)	NB (*n* = 3)	BCCDC (*n* = 2)	
Age (median, IQR)		62 (49–71)	58 (50–72)	59 (54–68)	54 (51–61)	54 (50–58)	61 (50–72)
Female sex (No. [%])		10 (34.5)	5 (29.4)	2 (25.0)	1 (33.3)	1 (50.0)	19 (32.2)
Clinical disease site^*b*^ (No. [%])	Central nervous system	7 (24.1)	5 (29.4)	1 (12.5)	1 (33.3)	0	14 (23.7)
	Pulmonary	9 (31.0)	4 (23.5)	1 (12.5)	1 (33.3)	2 (100)	17 (28.8)
	Cutaneous	0	0	0	0	0	0
	Disseminated (≥2 involved sites)	11 (37.9)	5 (29.4)	5 (62.5)	1 (33.3)	0	22 (37.3)
	Blood	0	1 (5.9)	1 (12.5)	0	0	2 (3.4)
	Other/unclear	2 (6.9)	2 (11.8)	0	0	0	4 (6.8)
Immunocompromised (No. [%])	Yes	19 (65.5)	9 (52.9)	3 (37.5)	2 (66.6)	1 (50.0)	34 (57.6)
	No	10 (34.5)	6 (35.3)	4 (50.0)	1 (33.3)	1 (50.0)	22 (37.3)
	Not available	0	2 (11.8)	1 (12.5)	0	0	3 (5.1)
HIV status (No. [%])	Positive	2 (6.9)	1 (5.9)	0	0	0	3 (5.1)
	Negative	27 (93.1)	13 (76.5)	6 (75.0)	3 (100)	1 (50.0)	50 (84.7)
	Not available	0	3 (17.6)	2 (25.0)	0	1 (50.0)	6 (10.2)
CSF findings^*c*^ (No. [%])	Elevated CSF protein	8 (27.6)	3 (17.6)	3 (37.5)	2 (66.6)	-	16 (27.1)
	Low CSF glucose	3 (10.3)	2 (11.8)	1 (12.5)	0	-	6 (10.2)
	Elevated WBC	10 (34.5)	2 (11.8)	3 (37.5)	2 (66.6)	-	17 (28.8)
	Lymphocytic predominance	8 (27.6)	1 (5.9)	3 (37.5)	1 (33.3)	-	13 (22.0)
	Neutrophilic predominance	2 (22.2)	1 (5.9)	0	1 (33.3)	-	4 (6.8)
Culture confirmation*^f^* (No. [%])	*Cryptococcus neoformans* s.l.	12 (41.4)	4 (23.5)	3 (37.5)	3 (100)	0	22 (37.3)
	*Cryptococcus gattii* s.l.	6 (20.7)	6 (35.3)	2 (25.0)	0	0	14 (23.7)
	Not available or negative fungal culture	11 (37.9)	7 (41.2)	3 (37.5)	0	2 (100)	23 (39.0)

^*a*^ BCCDC: British Columbia Centre for Disease Control; CSF: cerebrospinal fluid; HIV: human immunodeficiency virus; IH: Island Health; IHA: Interior Health Authority; IQR: inter-quartile range; NB: New Brunswick; No.: number; s.l.: sensu lato*.*

^*b*^ The clinical site of involvement is assumed to be isolated to the site unless listed as disseminated.

^*c*^ This group applies only to a subset of 36 individuals with cryptococcal meningitis. There were no cases of cryptococcal meningitis in the prospective study.

^*d*^ The IHA site is not included in this table as there were no individuals with cryptococcal disease.

^*e*^ One individual was included in both the retrospective and prospective cohorts.

^*f*^ As molecular genotyping to species or lineage level was available for only a subset of *Cryptococcus* isolates, results in this study are reported as sensu lato.

### Retrospective cohort

For the retrospective cohort only, the OPA between the FungiXpert and IMMY CrAg LFA for all sites was 96.0% (95% CI, 92.0%–98.4%), with a PPA of 92.0% (95% CI, 84.1%–96.7%), and NPA of 100% (95% CI, 95.9%–100.0%). Full performance characteristics by sample type and study site for the retrospective cohort are presented in Table 3. Across study sites, the OPA ranged from 92.8% to 100%, with PPA ranging from 85.7% to 100%, and NPA of 100%. Restricting the analysis to CSF samples, the OPA was 97.6% (95% CI, 91.8%–99.7%), with PPA of 93.9% (95% CI, 79.8%–99.3%) and NPA of 100% (95% CI, 93.1%–100.0%). Restricting the analysis to serum samples, the OPA was 94.5% (95% CI, 87.6%–98.2%), PPA was 90.7% (95% CI, 79.7%–96.9%), and NPA was 100% (95% CI, 90.6%–100.0%). A high titer sample was evaluated to assess for potential prozone effect; this sample demonstrated consistent detection at a titer of 1:2,560 across historical IMMY results and newly tested IMMY and FungiXpert results.

**TABLE 3:** Percent agreement for CrAg LFA qualitative detection for the retrospective cohort and for each site^*a*^

		Percent agreement (%)			Cohen’s Kappa (95% CI)
		Overall (95% CI)	Positive (95% CI)	Negative (95% CI)	
Full cohort	Overall	96.0 (92.0–98.4)	92.0 (84.1–96.7)	100 (95.9–100)	0.92 (0.86–0.98)
	CSF	97.6 (91.8–99.7)	93.9 (79.8–99.3)	100 (93.1–100)	0.95 (0.88–1)
	Serum	94.5 (87.6–98.2)	90.7 (79.7–96.9)	100 (90.6–100)	0.89 (0.79–0.98)
Site-specific	BCCDC	92.8 (84.9–97.3)	85.7 (71.5–94.6)	100 (91.4–100)	0.86 (0.75–0.97)
	FHA	100 (92.5–100)	100 (87.2–100)	100 (83.2–100)	1 (95% CI, 1–1)
	IH	94.1 (71.3–99.9)	90.9 (58.7–99.8)	100 (54.1–100)	0.88 (0.64–1)
	IHA	100 (83.9–100)	N/A^*b*^	100 (83.9–100)	N/A^*b*^
	NB^*c*^	100 (63.1–100)	100 (59.0–100)	100 (2.5–100)	1 (95% CI, 1–1)

^*a*^ CI: confidence interval; CSF: cerebrospinal fluid; IH: Island Health; IHA: Interior Health Authority; N/A: not available; NB: New Brunswick.

^*b*^ Analysis not performed due to lack of positive samples.

^*c*^ One sample considered concordant as nonreactive was subsequently reactive by FungiXpert testing at another laboratory and confirmed as a true reactive.

In addition, a total of eight samples were included for CrAg LA and FungiXpert CrAg LFA testing from the NB site, and were compared against both the IMMY and FungiXpert CrAg LFAs at the BCCDC site. These samples were contributed by three individuals with culture-positive cryptococcosis, all of which were identified as *C. neoformans* s.l. Of these, two results were discordant between CrAg LA testing and LFA testing (Table S1). These two samples were negative by LA testing, and one sample was positive by one of the two LFAs with a titer of 1:1, while the other sample was positive by both LFAs with a titer of 1:40.

A total of nine FungiXpert CrAg LFA results were identified as discrepant, of which two were CSF samples, and seven were serum samples. Of these nine samples, three were culture positive, including two positive for *C. neoformans* s.l. and one for *C. gattii* s.l. Eight results showed IMMY CrAg LFA positive and FungiXpert negative, with detected titers ranging from 1:1 to 1:80 (Table 4). In contrast, a single sample showed IMMY CrAg LFA negative and FungiXpert positive with a detected titer of 1:5 in an individual without known immunocompromise with culture-confirmed *C. neoformans* s.l. lung involvement (Table 4). Notably, this sample had been nonreactive by FungiXpert on initial testing and subsequently reproducibly reactive by FungiXpert at titers ranging from 1:1 to 1:5 at another laboratory.

**TABLE 4:** Discrepant cases for CrAg LFA qualitative and semi-quantitative results^*a*^

Cohort	Clinical characteristics	Sample type	Historical IMMY result (titer)	FungiXpert result (titer)	Orthogonal result	Final interpretation of FungiXpert result
Retrospective	58M, IC/HIV	Serum	Reactive (1:80)	Nonreactive	Disseminated cryptococcosis (CNS, skin, lung) diagnosed over 10 years prior with LP CrAg 1:1,024; persistently positive CrAg	False negative
	65F	Serum	Reactive (1:1)	Nonreactive	Fungal culture positive for *Cryptococcus neoformans* s.l. from BAL sample, same admission	False negative
	83F, IC/RA	Serum	Reactive (1:1)	Nonreactive	Fungal culture positive for *Cryptococcus gattii* s.l. from BAL sample 5 years prior	False negative
	47M	Serum	Nonreactive	Reactive^*b*^ (1:5)	Fungal culture positive for *Cryptococcus neoformans* s.l. from bronchoalveolar lavage fluid, same admission	True positive
	61M	CSF	Reactive (1:10)	Nonreactive	CSF India ink positive	False negative
	56M, IC/RA	CSF	Reactive (1:1)	Nonreactive	IMMY LFA CrAg positive (1:2,560) from serum	False negative
	62F, IC/RA	Serum	Reactive (1:5)	Nonreactive	Several earlier CrAg positive at 1:32	False negative
		Serum	Reactive (1:5)	Nonreactive		False negative
Prospective		Serum	Reactive (1:1)	Nonreactive		False negative

^*a*^ CNS: central nervous system; CrAg: cryptococcal antigen; CSF: cerebrospinal fluid; F: female; HIV: human immunodeficiency virus; IC: immunocompromised; LFA: lateral flow assay; LP: lumbar puncture; M: male; RA: rheumatoid arthritis.

^*b*^ This sample was nonreactive on initial FungiXpert testing. Repeat testing at another laboratory showed reactivity at a titer of 1:5. The result was reproducible with repeat titers ranging from 1:1 to 1:5.

**Fig 2:**
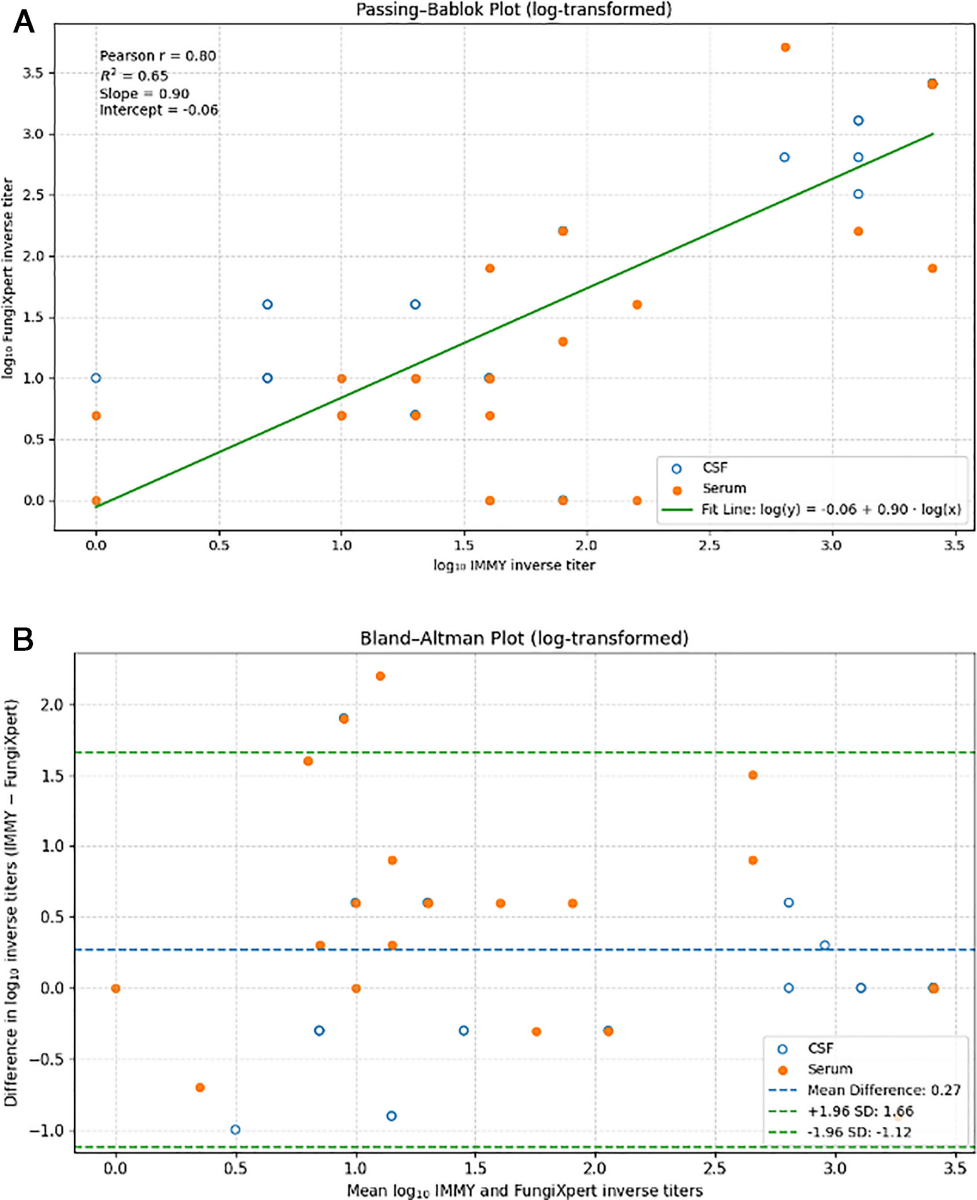
Method comparison of FungiXpert and IMMY CrAg LFAs. (**A**) Passing–Bablok regression plot of log₁₀-transformed inverse CrAg titers comparing IMMY and FungiXpert assays using combined retrospective and prospective samples. (**B**) Bland–Altman plot showing the difference in log₁₀ inverse titers (IMMY—FungiXpert) plotted against the mean of the two measurements. CSF samples are shown as blue open circles, and serum samples as solid orange circles. In panel B, the solid horizontal line represents the mean difference, and the dashed green lines represent the 95% limits of agreement (±1.96 standard deviations). The mean difference is above zero, indicating higher inverse titers measured by IMMY compared with FungiXpert. Greater dispersion is observed among serum samples, with several values exceeding the limits of agreement.

Semi-quantitative analysis was available for 54 positive samples overall tested at three sites. Of these, 30 (55.6%) FungiXpert titer results were within one doubling dilution of the original IMMY titer result, and the remaining 24 (44.4%) were two doubling dilutions or greater. Several discordant results were observed in serum samples at low titers (Table 4). The natural log–transformed inverse titers measured by the IMMY and FungiXpert CrAg LFAs were strongly correlated (Pearson r = 0.80; 95% CI, 0.65–0.88) (Fig. 2A). Bland–Altman analysis demonstrated a positive mean difference of 0.27 log units (95% limits of agreement, −1.12 to 1.66), indicating higher inverse titers with IMMY compared with FungiXpert (Fig. 2B).

### Prospective cohort

The prospective cohort included a total of 58 samples, with 50 serum samples (2 positive, 48 negative) and 8 CSF samples (0 positive, 8 negative). Percent agreement calculations were not performed owing to the low number of samples. The first positive serum sample was detected at a titer of 1:80 on IMMY and 1:1 on FungiXpert. The other positive serum sample was detected at a titer of 1:1 on IMMY and was not detected by FungiXpert (Table 4). All 56 negative samples by IMMY were also negative by FungiXpert.

### Analytical performance evaluation

#### Cross-reactivity

A total of 28 samples were tested for cross-reactivity across two sites, including primary clinical samples and culture suspensions from bacteria (*n* = 6), viruses (*n* = 13), fungi (*n* = 6), and samples from individuals with confirmed lymphoma (*n* = 3) (Table S2). Both the FungiXpert and IMMY assays cross-reacted with *Trichosporon asahii* culture suspension (confirmed by ITS fungal sequencing) at a titer of 1:160; otherwise, no cross-reactivity was observed.

#### Intra-assay precision (qualitative)

A total of five primary samples were selected for FungiXpert testing, including three serum samples and two CSF samples. The FungiXpert CrAg LFA testing showed that 15/15 (100%) positive sample results were reproducible across three consecutive days of testing in triplicate. In addition, five primary samples were included in the semi-quantitative intra-assay precision assessment, including two serum samples and three CSF samples. Nine results were generated for each sample, except for one CSF sample, which only had eight results due to insufficient sample volume. A total of 13/18 (72.2%) and 17/26 (65.4%) results were within one doubling dilution for serum and CSF samples, respectively, and all remaining results were within two doubling dilutions.

#### Inter-assay precision (qualitative and quantitative)

A total of five primary samples were selected for FungiXpert CrAg LFA testing, including two serum samples and three CSF samples across two reagent lots. Twelve results were generated for each serum sample, and six results were generated for each CSF sample, except for one sample, which only had five results due to insufficient sample volume (Table 5). All qualitative results were concordant. The serum samples demonstrated substantial variation by lot, with the widest difference observed with a serum sample with a titer range of 80–160 for lot 1, and 1,280–2,560 for lot 2 (Table 5). In contrast, all CSF sample titer results were within a doubling dilution of each other. In addition, a total of five primary samples were selected for IMMY CrAg LFA testing, including three serum samples and two CSF samples (Table S3). All qualitative results were concordant. The IMMY CrAg LFA semi-quantitative results showed the same titer for all results across the two reagent lots. Finally, the comparison of the repeat testing results to the historical IMMY results showed that all qualitative results were concordant, and one of the five samples showed a decrease in titer in a sample collected over 5 years prior to repeat testing (Table S3).

**TABLE 5:** Inter-assay precision for serum and CSF samples tested by the FungiXpert CrAg LFA^*a*^

Name	Sample type	Qualitative result	Lot 1^*b*^ Inv titer result	Lot 2^*c*^ Inv titer result	Difference in the number of doubling dilutions
1_1a	Serum	Reactive	5	5	Same
1_1b	Serum	Reactive	5	10	1
1_1c	Serum	Reactive	5	10	1
1_2a	Serum	Reactive	10	10	Same
1_2b	Serum	Reactive	10	10	Same
1_2c	Serum	Reactive	10	10	Same
2_1a	Serum	Reactive	80	1280	4
2_1b	Serum	Reactive	160	1280	3
2_1c	Serum	Reactive	160	1280	3
2_2a	Serum	Reactive	160	2560	5
2_2b	Serum	Reactive	80	1280	4
2_2c	Serum	Reactive	160	2560	5
3_1a	CSF	Reactive	5	10	1
3_1b	CSF	Reactive	5	10	1
3_1c	CSF	Reactive	5	10	1
4_1a	CSF	Reactive	80	40	1
4_1b	CSF	Reactive	80	40	1
4_1c	CSF	Reactive	80	40	1
5_1a	CSF	Reactive	320	640	1
5_1b	CSF	Reactive	320	640	1
5_1c	CSF	Reactive	NA	640	N/A

^*a*^ Inv: inverse; N/A: not available. The dark gray shade indicates a difference in doubling dilution of 3 or greater. The _1 and _2 notations refer to the day of testing.

^*b*^ FungiXpert Lot 1: CKS230919.

^*c*^ FungiXpert Lot 2: CKS231027.

## DISCUSSION

In this multicenter study, we evaluated the performance of the Health Canada-licensed FungiXpert CrAg LFA compared to the IMMY CrAg LFA across five healthcare facilities in two provinces in Canada. Our findings demonstrated comparable performance of FungiXpert CrAg for CSF samples; however, there was lower PPA for serum samples and substantial lot-to-lot variability with the FungiXpert CrAg LFA. This comparative diagnostic evaluation was motivated by the limited published data on the FungiXpert to support its clinical use, limited data for *Cryptococcus gattii* sensu stricto infection, and the context of a difference in regulatory status between the two assays. More specifically, the FungiXpert is currently licensed by Health Canada and not by the FDA, whereas the IMMY is currently licensed by the FDA and not Health Canada, which leads to different impacts on laboratory workflows for assay procurement.

In the overall study cohort, the test performance varied by sample type, with a PPA of 93.9% for CSF samples and PPA of 90.7% for serum samples. These results included a total of nine discrepant results, of which eight were missed detections by the FungiXpert CrAg LFA, where most occurred at low titers. However, there was a notable missed detection by FungiXpert CrAg LFA serum testing in an individual with HIV infection and an IMMY CrAg LFA titer of 1:80. This false-negative result is of particular concern given the potential clinical implications of a missed or delayed cryptococcosis diagnosis. High sensitivity of serum CrAg is essential to enable rapid diagnosis, leading to earlier initiation of antifungal therapy. In this study, we also noted several low serum titers (≤1:10). Although such low titers may be associated with false-positive results in individuals without prior history of cryptococcal infection (20), these results were deemed clinically compatible in all cases in our cohort. Furthermore, low serum CrAg titers may also be observed in asymptomatic infection or symptomatic diseases in both immunocompromised and immunocompetent hosts (21, 22). Interestingly, we also identified a single discrepant result in favor of FungiXpert CrAg LFA, where serum testing reproducibly detected a low titer (1:5–1:10) in an individual with respiratory culture-proven cryptococcal disease, confirming a true-positive result of the FungiXpert CrAg LFA.

Both assays demonstrated false-positive detection of *Trichosporon asahii* culture suspension, which is expected, given that both *Cryptococcus neoformans* and *Trichosporon asahii* express glucuronoxylomannan (23) and is consistent with earlier evaluation of both assays (15). The clinical significance of this cross-reactivity is limited due to the rarity of this infection; however, invasive infection may overlap clinically, especially in individuals with hematological disorders, such that further diagnostic differentiation may be required (24).

Inter-assay precision evaluation highlighted substantial variability in the FungiXpert CrAg LFA results for the two different reagent lots evaluated. More specifically, nearly one-third of the samples evaluated for inter-lot reproducibility demonstrated ≥3 doubling dilution difference between two FungiXpert LFA lots, which may complicate the interpretation of follow-up titer results for clinical monitoring. However, there was no difference between lots for qualitative detection across these same lots. Furthermore, this precision assessment was based on a low number of clinical samples due to limited sample availability and should be investigated further. These findings highlight concern from the laboratory perspective; however, based on recently updated global guidelines for the management of cryptococcosis, use of CrAg titers longitudinally is not recommended for clinical decision-making (25).

Several variables need to be considered in the selection of cryptococcal LFA testing, including regulatory status of the assay, test performance characteristics, cost, and supply chain considerations. In our Canadian study setting, the cost of testing reagents for both assays was similar, with each estimated at 11 CAD/sample. However, as highlighted earlier, the differences in regulatory status impact laboratory workflow and the sustainability of the supply chain process for testing. Given that the IMMY assay is not currently licensed by Health Canada, procurement of IMMY kits for routine clinical testing must be obtained through the Health Canada Special Access Program, which typically requires a monthly application with supporting paperwork. This procurement pathway is not a sustainable option and represents an administrative burden (26, 27). The current study provides valuable comparative test performance data, including the finding of a performance gap for serum with the currently approved Health Canada FungiXpert assay, which highlights an unmet need, and the decision to request licensing for an assay, including for the IMMY CrAg LFA, is at the discretion of the vendor. In addition, the importance of environmental sustainability in clinical diagnostic evaluation is increasingly recognized (28); in this study, both CrAg LFA assays evaluated generated similar waste.

Our findings can be contrasted with a prior single-site retrospective study from China evaluating 199 samples from 158 patients, which reported high diagnostic performance of the FungiXpert CrAg LFA compared with the IMMY CrAg LFA (99.1% sensitivity and 98.9% specificity) (15). Nearly 70% of individuals in that cohort had cryptococcal meningitis, and only one discrepant result was identified, consisting of a low-titer CSF sample (1:2) adjudicated as a false-negative FungiXpert result (15). Although overall agreement was high, Liu et al. observed variability in semi-quantitative titers, with FungiXpert yielding equal or higher titers than IMMY in approximately 40% of samples. In contrast, our regression and Bland–Altman analyses demonstrated strong linear agreement between assays but identified a small positive mean difference favoring higher inverse titers measured by IMMY. These differences may reflect variation in study design, patient population, disease severity, and analytical approach, as well as the lack of stratification by specimen type or titer range in the earlier study.

This study was strengthened by its multi-site nature, sample size, and comprehensive evaluation of test performance, including cross-reactivity and precision. However, several limitations should be acknowledged. The retrospective nature of a significant portion of our data cannot properly account for differences in sample handling and storage conditions, which may have confounded result interpretation and for missing clinical metadata. However, a limited sample stability study performed within this project demonstrated results robust to the effect of time and freeze-thawing, which would limit this impact. Furthermore, semi-quantitative results were only partially available for some sites, restricting a comprehensive quantitative comparison across all cohorts. In addition, owing to limited samples, we were only able to characterize one high-titer sample to assess the prozone effect. This will require further study with a greater number of high-burden samples to fully characterize. We were unable to characterize the full distribution of *Cryptococcus neoformans* vs *Cryptococcus gattii* species complex, given the incomplete culture confirmation, and which would be of particular interest in our study setting. Furthermore, we did not investigate the test performance of other pathogenic members within the *C. gattii/neoformans* complex, such as *C. deneoformans, C. bacillisporus,* and *C. tetragattii,* in this study, and genetic diversity within cryptococcal species has been recognized to potentially affect test performance (29). Additional testing to evaluate cross-reactivity with *Paracoccidioides brasiliensis* would also be of value and could not be included in the current study due to a lack of material. These limitations may have affected the generalizability of our findings and highlight the need for more comprehensive future prospective studies.

In summary, this study extended our understanding of the performance of the FungiXpert CrAg LFA in a real-world, multicenter setting in Canada, including BC, which is endemic for cryptococcal infection. The assay demonstrated reasonable agreement for CSF, but lower agreement for serum samples, which increases concern for false-negative results. Substantial lot-to-lot variability was also noted. These findings, along with assay regulatory licensing status, should be considered in the decision for clinical implementation of CrAg LFA testing.

## SUPPLEMENTAL MATERIAL

Supplemental material jcm.01438-25-s0001.docx Supplemental material and Tables S1 to S3.

ASM does not own the copyrights to Supplemental Material that may be linked to, or accessed through, an article. The authors have granted ASM a non-exclusive, world-wide license to publish the Supplemental Material files. Please contact the corresponding author directly for reuse.
