# Supplementary material for: Test performance of a commercial cryptococcal antigen lateral flow assay: a retrospective and prospective study at five Canadian sites
Source: J Clin Microbiol. 2026 Apr 22;64(5):e01438-25. doi: 10.1128/jcm.01438-25 (PMC13170270; doi:10.1128/jcm.01438-25)
Supplement: Supplemental material — and Tables S1 to S3. [file jcm.01438-25-s0001.docx]

**Supplemental Material**

*Culture suspension protocol*

1. A 0.5 McFarland concentration of *Cryptococcus gattii* s.l. (clinical isolate), *Candida albicans* (clinical isolate), *Trichosporon* *asahii* (clinical isolate), and *Streptococcus pneumoniae* (ATCC 49619) was generated by the BCCDC PHL using pure culture isolate and 0.85% saline for dilution.
2. A total of 50uL was used for the positive, negative and sample treatment solution volumes instead of drops.
3. Follow kit package insert for screening test procedure using the 0.5 McFarland concentration directly as the sample.
4. Perform all tests in glass test tubes as per established CrAg LFA procedure.

**Supplemental Table 1.** Subset of samples tested against *Cryptococcus* latex agglutination at one site.

|  | **NB** | | | | | **BCCDC** | | | |
| --- | --- | --- | --- | --- | --- | --- | --- | --- | --- |
| **ID** | **Sample Type** | **Cryptolatex** | **Cryptolatex titer** | **FungiXpert** | **FungiXpert Inverse titer** | **FungiXpert** | **FungiXpert Inverse titer** | **BCCDC IMMY** | **BCCDC IMMY Inverse titer** |
| NB1 | CSF | reactive | 8,192 | reactive | >2,560 | reactive | >2,560 | reactive | >2,560 |
| NB2 | CSF | NR | NR | reactive | 40 | reactive | 40 | reactive | 20 |
| NB3 | Serum | reactive | 1,024 | reactive | >2,560 | reactive | >2,560 | reactive | >2,560 |
| NB4 | Serum | reactive | 8 | reactive | 40 | reactive | 160 | reactive | 160 |
| NB5 | Serum | reactive | 8 | reactive | 40 | reactive | 80 | reactive | 160 |
| NB6 | Serum | reactive | 8 | reactive | 20 | reactive | 80 | reactive | 80 |
| NB7 | Serum | reactive | 8 | reactive | 20 | reactive | 80 | reactive | 80 |
| NB8 | Serum | NR | N/A | NR | N/A | reactive | 1* | NR | N/A |

INV: inverse; N/A: not available; NB: New Brunswick; NR: nonreactive.

*Result was reproducible with repeat titer of 5 and 1.

**Supplemental Table 2.** Distribution of samples tested for cross-reactivity with the FungiXpert and IMMY CrAg lateral flow assays.

| **Group** | **Organism** | **Testing site** | **Sample type** | **IMMY result** | **FungiXpert result** |
| --- | --- | --- | --- | --- | --- |
| Virus  (*n*=13) | VZV | IHA | CSF | NR | NR |
|  | VZV | IHA | CSF | NR | NR |
|  | VZV | IHA | CSF | NR | NR |
|  | mumps | BCCDC | serum | NR | NR |
|  | mumps | BCCDC | serum | NR | NR |
|  | HBV | BCCDC | serum | NR | NR |
|  | HBV | BCCDC | serum | NR | NR |
|  | HCV | BCCDC | serum | NR | NR |
|  | HCV | BCCDC | serum | NR | NR |
|  | HIV | BCCDC | serum | NR | NR |
|  | HIV | BCCDC | serum | NR | NR |
|  | HIV | BCCDC | serum | NR | NR |
|  | HIV | BCCDC | serum | NR | NR |
| Bacterial  (*n*=6) | *S. pneumoniae* | BCCDC | culture suspension | NR | NR |
|  | *Borrelia burgdorferi* | BCCDC | serum | NR | NR |
|  | *Borrelia burgdorferi* | BCCDC | serum | NR | NR |
|  | *Mycobacterium tuberculosis* | BCCDC | serum | NR | NR |
|  | *Mycobacterium tuberculosis* | BCCDC | serum | NR | NR |
|  | *Mycobacterium tuberculosis* | BCCDC | serum | NR | NR |
| Fungal  (*n*=6) | *Candida albicans* | BCCDC | culture suspension | NR | NR |
|  | *Trichosporon*  *asahii* | BCCDC | culture suspension | reactive (1:160) | reactive (1:160) |
|  | *Trichosporon*  *asahii* | BCCDC | culture suspension | reactive (1:160) | reactive (1:160) |
|  | galactomannan reactive | BCCDC | serum | NR | NR |
|  | galactomannan reactive | BCCDC | serum | NR | NR |
|  | galactomannan reactive | BCCDC | serum | NR | NR |
| Non-infectious | lymphoma | IHA | serum | NR | NR |
|  | lymphoma | IHA | serum | NR | NR |
|  | lymphoma | IHA | serum | NR | NR |

BCCDC: British Columbia Centre for Disease Control; CSF: cerebrospinal fluid; IHA: Interior Health Authority; NR: non reactive; spp.: species.

**Supplemental Table 3.** Historical IMMY results and inter-assay precision for serum and CSF samples tested by the IMMY CrAg LFA.

| **Name** | **Sample type** | **Qualitative result** | **Historical**  **Inverse titer result** | **Lot 1***  **Inverse titer result** | **Lot 2****  **Inverse titer result** | **Difference in doubling dilutions between lot 1 and lot 2** |
| --- | --- | --- | --- | --- | --- | --- |
| 1_1a | serum | reactive | 40 | 40 | 40 | same |
| 2_1a | serum | reactive | 160 | 160 | 160 | same |
| 3_1a | serum | reactive | 2560 | 2560 | 2560 | same |
| 4_1a | CSF | reactive | 20 | 20 | 20 | same |
| 5_1a^#^ | CSF | reactive | 1280 | 160 | 160 | same |

CSF: cerebrospinal fluid.

*IMMY Lot 1: F1011242

**IMMY Lot 2: F1011248

^#^Overall, as presented in the table, samples demonstrated stable IMMY CrAg LFA results over time. However, this sample was collected over 5 years prior to retesting with lots 1 and 2. Sample degradation may have occurred to explain the difference in inverse titer result between historical and repeat testing results.
